# Inoculation for Cholera and Yellow Fever

**Published:** 1885-07

**Authors:** 


INOCULATION FOR CHOLERA AND YELLOW FEVER.

Telegraphic despatches to the daily papers announce brilliant results from the inoculation experiments of DR. FERRAN, of Valencia, Spain. It is stated that over six thousand persons have been inoculated with cholera microbes, as a prophylactic, in the province of Valencia.

A despatch, dated, Soudan, 29th June, contains the following words :

"The Spanish Admiralty authorities have ordered the cholera vaccination system of Dr. Ferran to be applied to all the officers and men of the Royal Navy."

Professor H. C. Wood makes the following terse comment upon the subject in the *Therapeutic Gazette*, 15th of June, 1885.

"While the system is said to be entirely successful, we cannot see on what grounds that statement is based, as it is far too early in the season to judge of the severity of an epidemic, and no proof can yet be offered as to the immunity of the inoculated individuals from cholera. Before we can indulge our enthusiasm we must demand many further details."

DR. CARMONA is experimenting in inoculation with yellow fever germs in Mexico. Subjects are not wanting, but the experiments are apparently not so successful as those of Dr. Ferran.

"The germ in Mexico and in yellow-fever does not prove as tractable as in Spain and in cholera."

